# Supplementary material for: Patterns and ecological drivers of viral communities in acid mine drainage sediments across Southern China
Source: Nat Commun. 2022 May 2;13:2389. doi: 10.1038/s41467-022-30049-5 (PMC9061769; doi:10.1038/s41467-022-30049-5)
Supplement: Supplementary file 1 — Supplementary Information [file 41467_2022_30049_MOESM1_ESM.pdf]

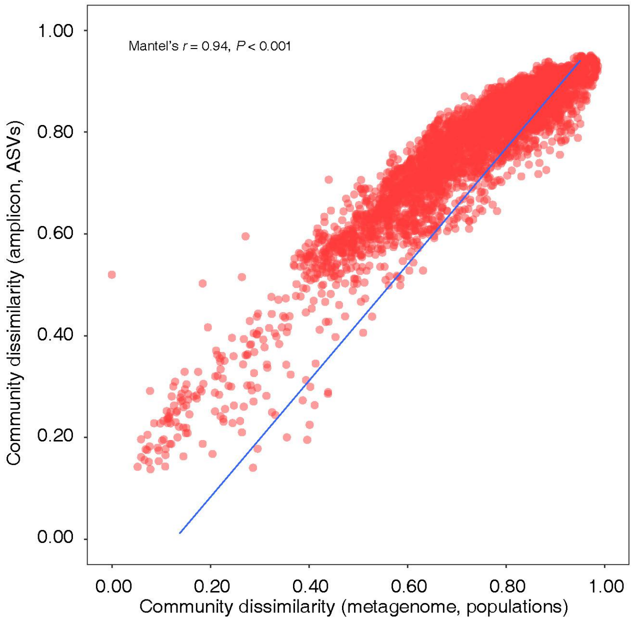

Supplementary Fig. 1 | Similarities between prokaryotic communities recovered from metagenome and amplicon sequencing. Mantel's correlation coefficients were used to represent the similarity between Bray-Curtis dissimilarity of community compositions evaluated by amplicons sequence variants (ASVs) applied in previous study[1] and population genomes in this study. The statistical test used was two-tailed. Source data are provided in the Source Data file.

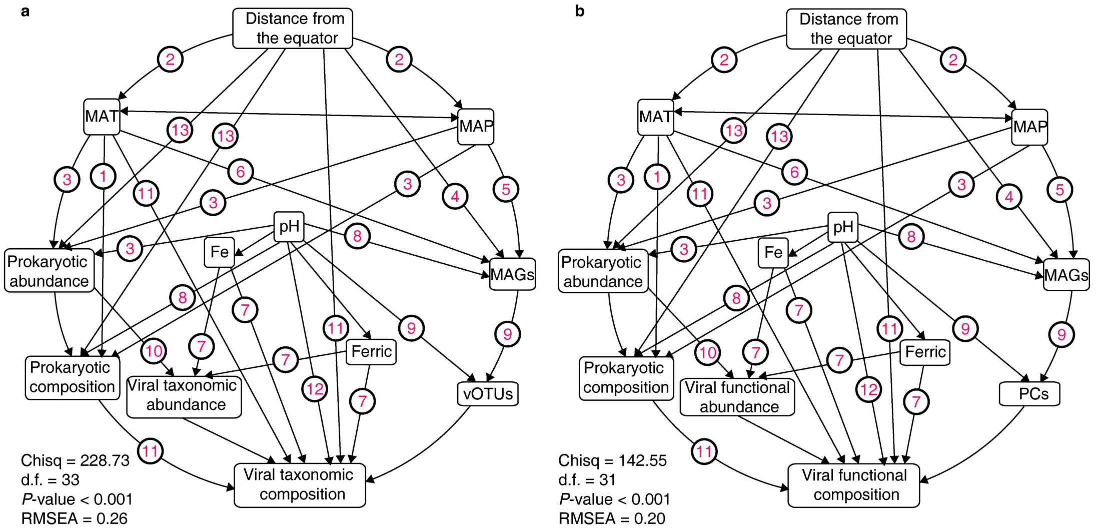

Supplementary Fig. 2| Priori structural equation models (SEM) used in this study. Our models evaluated the effects of biotic or abiotic factors on viral taxonomic (**a**) and functional (**b**) communities. The numbers in the arrows denote example references used to support our predictions (see Supplementary references below). Arrows without numbers indicate theoretical predictions. The fit of models was evaluated using one-tailed Chi-squared test and root mean square error of approximation (RMSEA). Source data are provided in the Source Data file.

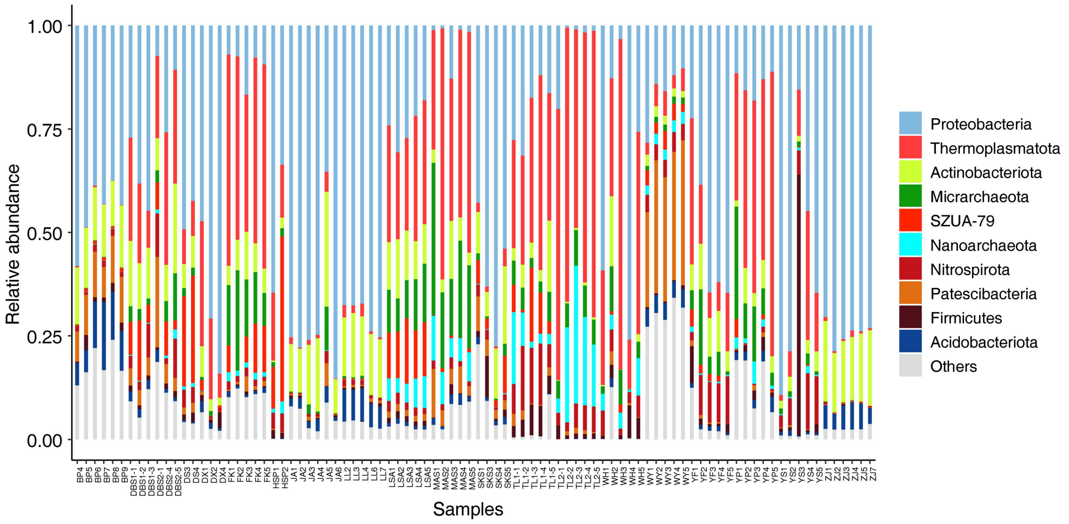

Supplementary Fig. 3 | Composition of prokaryotic community. The bar graphs indicate relative abundance of prokaryotic populations (coloured by phyla) in the 90 acid mine drainage (AMD) sediments revealed by metagenomic sequencing. Source data are provided in the Source Data file.

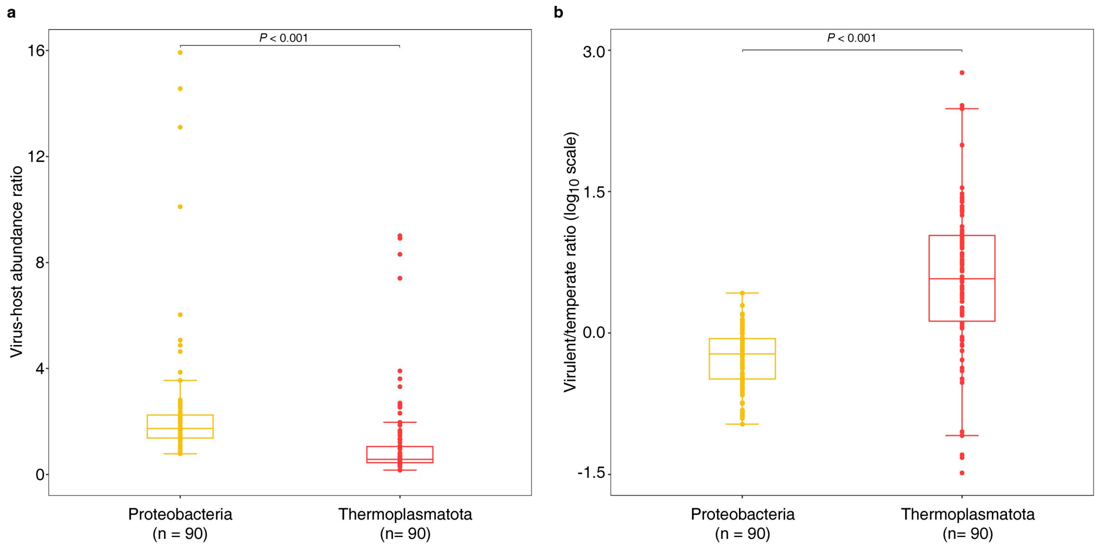

Supplementary Fig. 4 | Boxplot indicate virus/host abundance ratios (VHRs) (**a**) and virulent/temperate abundance ratios (**b**) in *Proteobacteria* (n = 90 biologically independent samples) and *Thermoplasmatota* (n = 90 biologically independent samples). Bartlett's test for equal variance and Shapiro-Wilk normality test for normality between the two groups of VHRs was significant at the 1% level. Two-tailed *P*-values from non-parametric Wilcoxon *t*-test (unpaired) are indicated in **a**, **b**. Horizontal lines within the boxes indicate median values, the boxes indicate the interquartile range, whiskers show the smallest and largest values within 1.5 times the interquartile ranges above and below the 75th and 25th percentile, and dots represent VHRs (**a**) and virulent/temperate abundance ratios (**b**) in the sediment samples. Source data are provided in the Source Data file.

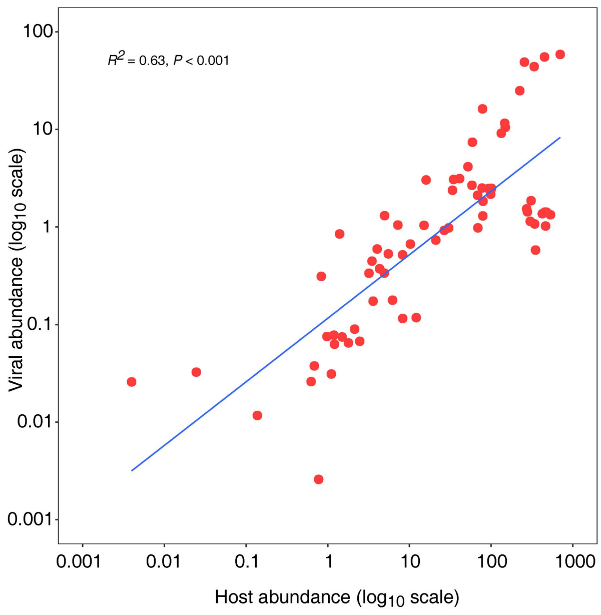

Supplementary Fig. 5 | Linear regression relationship between the abundance of the provirus encoding *phnCDE* genes, and the abundance of its host populations. Best-fit lines and adjusted R^2 values for the linear regression are presented. The statistical test used was two-tailed. Source data are provided in the Source Data file.

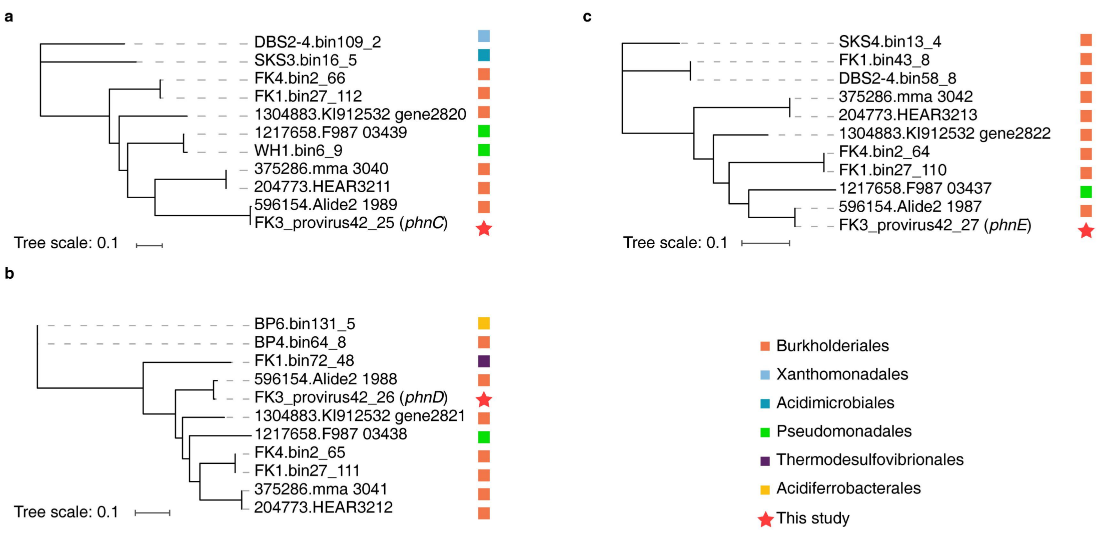

Supplementary Fig. 6 | Maximum-likelihood phylogenetic tree with *phnCDE* genes from AMD sediments (indicated by stars) compared to homologs found in eggNOG v5.0.0 database and the host proteins coloured by different orders indicated by different colours of squares. Source data are provided in the Source Data file.

Supplementary references

1. Hao, Y. Q. et al. Microbial biogeography of acid mine drainage sediments at a regional scale across Southern China. *FEMS Microbiol. Ecol.* **98**, fiac002 (2022).
2. Hijmans, R. J. et al. Very high resolution interpolated climate surfaces for global land areas. *Int. J. of Climatol.* **25**, 1965-1978 (2005).
3. Bahram, M. et al. Structure and function of the global topsoil microbiome. *Nature* **560**, 233-237 (2018).
4. Lyngwi, N. A., Koijam, K., Sharma, D. & Joshi, S. R. Cultivable bacterial diversity along the altitudinal zonation and vegetation range of tropical Eastern Himalaya. *Rev Biol Trop.* **61**, 467-90 (2013).
5. Delgado-Baquerizo, M. et al. Microbial diversity drives multifunctionality in terrestrial ecosystems. *Nat Commun*. **7**, 10541 (2016).
6. Nottingham, A. T. et al. Microbes follow Humboldt: temperature drives plant and soil microbial diversity patterns from the Amazon to the Andes. *Ecology* **99**, 2455-2466 (2018).
7. Bonnain, C., Breitbart, M. & Buck, K. N. The Ferrojan horse hypothesis: iron-virus interactions in the ocean. *Front. Mar. Sci.* **3**, 82 (2016).
8. Kuang, J. L. et al. Contemporary environmental variation determines microbial diversity patterns in acid mine drainage. *ISME J.* **7**, 1038-1050 (2013).
9. Gao, S. M. et al. Depth-related variability in viral communities in highly stratified sulfidic mine tailings. *Microbiome* **8**, 89 (2020).

10. Li, Z. et al. Deep sea sediments associated with cold seeps are a subsurface reservoir of viral diversity. *ISME J.* **15**, (2021).

11. Brum, J. R. et al. Patterns and ecological drivers of ocean viral communities. *Science* **348**, 1261498 (2015).

12. Adriaenssens, E. M. et al. Environmental drivers of viral community composition in Antarctic soils identified by viromics. *Microbiome* **5**, 83 (2017).

13. Siles, J. A., Cajthaml, T., Minerbi, S. & Margesin, R. Effect of altitude and season on microbial activity, abundance and community structure in Alpine forest soils. *FEMS Microbiol Ecol.***92**, fiw008 (2016).
