# Supplementary material for: Patterns and ecological drivers of viral communities in acid mine drainage sediments across Southern China
Source: Nat Commun. 2022 May 2;13:2389. doi: 10.1038/s41467-022-30049-5 (PMC9061769; doi:10.1038/s41467-022-30049-5)
Supplement: Supplementary file 2 — Reporting Summary [file 41467_2022_30049_MOESM2_ESM.pdf]

nature portfolio

Corresponding author(s): Linan Huang
Last updated by author(s): Apr 1, 2022

Reporting Summary

Nature Portfolio wishes to improve the reproducibility of the work that we publish. This form provides structure for consistency and transparency in reporting. For further information on Nature Portfolio policies, see our Editorial Policies and the Editorial Policy Checklist.

Statistics

For all statistical analyses, confirm that the following items are present in the figure legend, table legend, main text, or Methods section.

n/a	Confirmed	
☐	☒	The exact sample size (n) for each experimental group/condition, given as a discrete number and unit of measurement
☐	☒	A statement on whether measurements were taken from distinct samples or whether the same sample was measured repeatedly
☐	☒	The statistical test(s) used AND whether they are one- or two-sided<br>*Only common tests should be described solely by name; describe more complex techniques in the Methods section.*
☒	☐	A description of all covariates tested
☐	☒	A description of any assumptions or corrections, such as tests of normality and adjustment for multiple comparisons
☐	☒	A full description of the statistical parameters including central tendency (e.g. means) or other basic estimates (e.g. regression coefficient) AND variation (e.g. standard deviation) or associated estimates of uncertainty (e.g. confidence intervals)
☐	☒	For null hypothesis testing, the test statistic (e.g. F, t, r) with confidence intervals, effect sizes, degrees of freedom and P value noted<br>*Give P values as exact values whenever suitable.*
☒	☐	For Bayesian analysis, information on the choice of priors and Markov chain Monte Carlo settings
☒	☐	For hierarchical and complex designs, identification of the appropriate level for tests and full reporting of outcomes
☐	☒	Estimates of effect sizes (e.g. Cohen's d, Pearson's r), indicating how they were calculated

Our web collection on statistics for biologists contains articles on many of the points above.

Software and code

Policy information about availability of computer code

Data collection	eggNOG v5.0.0<br>NCBI Viral RefSeq v201<br>WorldClim v2.1
Data analysis	Abawaca v1.0.0, BamM v1.7.3, Bowtie2, CheckM v1.1.3, CheckV v0.6.0, Concoct v0.4.0, CoverM v0.6.0, DADA2 v1.16.0, DASTool v1.1.2, DeePhage v1.0, GTDB-Tk v1.6.0, iqtree v2, MATLAB BiMat package, MaxBin v2.2.2, MetaBAT v2.12.1, Muscle v3.8.31, Prodigal v2.6.3, R v4.0.3, RefineM v0.0.24, SPAdes v3.14.1, TrimAL v1.4.rev22, VirSorter v1.0.6 and vConTACT v2.0. Custom codes were used as explained at https://github.com/eco-gaoshaom/viral-biogeography (https://doi.org/10.5281/zenodo.6374561).

For manuscripts utilizing custom algorithms or software that are central to the research but not yet described in published literature, software must be made available to editors and reviewers. We strongly encourage code deposition in a community repository (e.g. GitHub). See the Nature Portfolio guidelines for submitting code & software for further information.

Data

Policy information about availability of data

All manuscripts must include a data availability statement. This statement should provide the following information, where applicable:

- Accession codes, unique identifiers, or web links for publicly available datasets
- A description of any restrictions on data availability
- For clinical datasets or third party data, please ensure that the statement adheres to our policy

Data availability. Raw reads of metagenomes and all assembled prokaryotic population genomes have been deposited in NCBI BioProject database https://www.ncbi.nlm.nih.gov/bioproject/PRJNA666025. Short Reads Archive accession numbers for individual reads are listed in Supplementary Data 8. Biosample

accession numbers for individual prokaryotic genomes are listed in Supplementary Data 9. Assembled viral genomes are available from the NCBI BioProject database https://www.ncbi.nlm.nih.gov/bioproject/PRJNA648034. eggNOG database is available at http://eggnog5.embl.de/download/eggnog_5.0/. NCBI viral RefSeq database is available at https://ftp.ncbi.nlm.nih.gov/refseq/release/. WorldClim database is available at https://www.worldclim.org/data/worldclim21.html. Source data are provided with this paper.

Field-specific reporting

Please select the one below that is the best fit for your research. If you are not sure, read the appropriate sections before making your selection.

☐ Life sciences ☐ Behavioural & social sciences ☒ Ecological, evolutionary & environmental sciences

For a reference copy of the document with all sections, see nature.com/documents/nr-reporting-summary-flat.pdf

Ecological, evolutionary & environmental sciences study design

All studies must disclose on these points even when the disclosure is negative.

Study description

AMD environments are ideal targets for quantitative, genomic-based analyses of microbial ecology and evolution and community function due to their geochemical and biological simplicity, and broad and steep environmental gradients. We conducted metagenomic sequencing on acid mine drainage (AMD) sediments (n = 90) sampled across Southern China. The viral and prokaryotic genomes recovered from metagenomes were analysed with geographic location, climate data, and geochemical variables for each sample to determine whether viral populations and functions exhibit specific biogeographic patterns in the context of variations in biotic and abiotic factors.

Research sample

The AMD sediment samples were collected from 18 geochemically diverse mine sites from six provinces across Southern China. The choice of the 18 mine sites is due to the fact that AMD is typical feature in these mining areas, based on the information reported from previous studies or our field investigations specially for the current study. Sediments were collected for each site, and metagenome was generated, geographic location recorded, and geochemical parameters measured for each sample in order to analyse the local and regional distribution patterns and ecological drivers of viral communities.

Sampling strategy

Samples were collected using a shovel from the top 10 cm of AMD sediments either at the centre or at ~1m from the edge of AMD ponds depending on the safety and size of the features at each mine site. The samples were sealed in 50 mL sterile tubes, kept in an icebox and transported to the laboratory, where they were stored at 4 °C and processed within 24 h. Each sediment was well mixed and divided into two fractions: one fraction for DNA extraction (stored at -80 °C) and the other for physicochemical measurements (air-dried). The choice of the depth (10 cm) is due to the fact that the microbial abundance decreased sharply at greater depth [1]. The choice of 50 mL sterile tubes is due to the fact that sediment used for the measurements of geochemical parameters is no more than 25 g and for the DNA extraction is 10 g (Methods section in the main manuscript).

[1] Sánchez-Andrea et al., Appl. Environ. Microbiol. 78, 4638-4645 (2012).

Data collection

The metagenomic library was prepared by S.M.G. and sequenced on an Illumina MiSeq platform (150 bp, paired end reads). Sequencing reads were processed by S.M.G. for quality control, scaffolds assembly, genome binning, and viral sequence identification.

Timing and spatial scale

All samples were collected during Aug-2017 to Oct-2017 across Southern China. Sediments were collected in different months due to the overall time needed to sample the 18 mine sites (i.e., traveling, site surveying, and sampling). Sediments from the mine sites of BP, DS, HSP, JA, LL, LSA, SKS, WY, and ZJ were collected in Aug-2017. Sediments from DBS, FK, YF, YP, and YS were collected in Sep-2017. Sediments from MAS, TL, and WH were collected in Oct-2017.

Data exclusions

Sediment samples with low DNA yield/quality were discarded.

Reproducibility

This experiment has not been reproduced but can be reproduced with the methods and supplementary data and codes provided in the manuscript.

Randomization

The mine sites with AMD were randomly distributed across Southern China. Besides, all samples were randomly collected depending on the safety and size of the features at each mine site.

Blinding

Blinding was not applicable to this study, as our analyses used multiple publicly available databases collected prior to this study.

Did the study involve field work? ☒ Yes ☐ No

Field work, collection and transport

Field conditions

Site locations and characteristics of the AMD sediment samples were provided in our previous study which is cited in our manuscript as ref. 24. Overall, the mean annual temperature of the sampling sites ranged between 14.0 - 20.9 °C, and the mean annual precipitation ranged between 1,110 - 1,849 mm. Besides, environmental conditions of the sampled AMD sediments were highly variable. For example, the pH values ranged from 1.46 to 6.80, and total Fe concentration ranged from 15.9 to 387.8 mg/g while ferric iron concentration ranged from 0.09 to 33.1 mg/g. Detailed environmental parameters could be found in the Source Data provided alongside the main manuscript.

Location

Location information is available in Fig. 1a. Specifically, these samples were distributed from the Guizhou, Guangdong, Guangxi, Jiangxi, Hunan and Anhui provinces across Southern China (22.96°-31.68°N, 105.73°-118.63°E). Detailed latitude and longitude for

	each sample are available in the provided Source Data file.
Access & import/export	There was no need for any application to collect AMD sediments in China. The import/export statement was not relevant to our samples because they were transported and processed in China.
Disturbance	No disturbances were caused by this study.

Reporting for specific materials, systems and methods

We require information from authors about some types of materials, experimental systems and methods used in many studies. Here, indicate whether each material, system or method listed is relevant to your study. If you are not sure if a list item applies to your research, read the appropriate section before selecting a response.

Materials & experimental systems

n/a	Involved in the study	
☒	☐	Antibodies
☒	☐	Eukaryotic cell lines
☒	☐	Palaeontology and archaeology
☒	☐	Animals and other organisms
☒	☐	Human research participants
☒	☐	Clinical data
☒	☐	Dual use research of concern

Methods

n/a	Involved in the study	
☒	☐	ChIP-seq
☒	☐	Flow cytometry
☒	☐	MRI-based neuroimaging
