# Supplementary material for: Patterns and ecological drivers of viral communities in acid mine drainage sediments across Southern China
Source: Nat Commun. 2022 May 2;13:2389. doi: 10.1038/s41467-022-30049-5 (PMC9061769; doi:10.1038/s41467-022-30049-5)
Supplement: Supplementary file 3 — Description of Additional Supplementary data 1-9 [file 41467_2022_30049_MOESM3_ESM.pdf]

Title: Supplementary Data 1
Description: Detailed information of predicted viral genomes identified in the 90 acid mine drainage (AMD) sediment samples.

Title: Supplementary Data 2
Description: Biotic data for the 90 AMD sediment samples across Southern China. OTUs, operational taxonomic units; PCs, protein clusters; MAGs, metagenomic assembled genomes.

Title: Supplementary Data 3
Description: Abundance and taxonomic affiliation of the dereplicated viral populations.

Title: Supplementary Data 4
Description: Virus-host linkages predicted by prophages, shared genomic matches with host genomes, and protospacer-spacer matches.

Title: Supplementary Data 5
Description: Detailed function descriptions and sequence information of the viral genomes containing *phoH* and *phnCDE* genes.

Title: Supplementary Data 6
Description: Homologs of viral *phoH*, *phnC*, *phnD*, and *phnE* genes recruited from eggNOG v5.0.0 database and prokaryotic genomes.

Title: Supplementary Data 7
Description: Abundance and taxonomic affiliation of the dereplicated prokaryotic populations.

Title: Supplementary Data 8
Description: Detailed accession number of metagenomic and amplicon reads for the 90 sediment samples.

Title: Supplementary Data 9
Description: Detailed accession number of metagenome-assembled population genomes reconstructed from the 90 sediment samples.
